# Ectopic mineralization of cartilage and collagen-rich tendons and ligaments in *Enpp1^asj-2J^* mice

**DOI:** 10.18632/oncotarget.7455

**Published:** 2016-02-17

**Authors:** Jieyu Zhang, Nathaniel A. Dyment, David W. Rowe, Sarah Y. Siu, John P. Sundberg, Jouni Uitto, Qiaoli Li

**Affiliations:** ^1^ Department of Dermatology and Cutaneous Biology, The Sidney Kimmel Medical College at Thomas Jefferson University, Philadelphia, PA, USA; ^2^ Department of Dermatology, The Fourth Military Medical University, Xijing Hospital, Xi'an, China; ^3^ Center for Regenerative Medicine and Skeletal Development, University of Connecticut Health Center, Farmington, CT, USA; ^4^ The Jackson Laboratory, Bar Harbor, ME, USA

**Keywords:** ectopic mineralization, tendon and ligament calcification, chondrocalcinosis, generalized arterial calcification of infancy, mouse models, Pathology Section

## Abstract

Generalized arterial calcification of infancy (GACI), an autosomal recessive disorder caused by mutations in the *ENPP1* gene, manifests with extensive mineralization of the cardiovascular system. A spontaneous *asj-2J* mutant mouse has been characterized as a model for GACI. Previous studies focused on phenotypic characterization of skin and vascular tissues. This study further examined the ectopic mineralization phenotype of cartilage, collagen-rich tendons and ligaments in this mouse model. The mice were placed on either control diet or the ‘acceleration diet’ for up to 12 weeks of age. Soft connective tissues, such as ear (elastic cartilage) and trachea (hyaline cartilage), were processed for standard histology. Assessment of ectopic mineralization in articular cartilage and fibrocartilage as well as tendons and ligaments which are attached to long bones were performed using a novel cryo-histological method without decalcification. These analyses demonstrated ectopic mineralization in cartilages as well as tendons and ligaments in the homozygous *asj-2J* mice at 12 weeks of age, with the presence of immature osteophytes displaying alkaline phosphatase and tartrate-resistant acid phosphatase activities as early as at 6 weeks of age. Alkaline phosphatase activity was significantly increased in *asj-2J* mouse serum as compared to wild type mice, indicating increased bone formation rate in these mice. Together, these data highlight the key role of ENPP1 in regulating calcification of both soft and skeletal tissues.

## INTRODUCTION

Ectopic mineralization, characterized by deposition of calcium hydroxyapatite complexes on soft connective tissues, is a common age-associated phenomenon. Depending on the inflicted tissue, these acquired pathological processes can cause considerable morbidity [1]. Ectopic mineralization of cartilage and collagen-rich tendons and ligaments can be a cause of considerable orthopedic problems resulting in limitations in mobility due to tissue degeneration, and often requiring medical or surgical intervention [2, 3]. Mechanistically, two major types of ectopic mineralization processes have been recognized [4]. First, metastatic calcification refers to deposition of calcium complexes as a result of elevated serum levels of calcium and/or phosphate, while dystrophic calcification is secondary to some form of insult to the tissues, frequently observed as a result of trauma and inflammatory diseases. In dystrophic calcification, serum calcium/phosphate levels are normal. Thus, ectopic mineralization can represent the consequences of several contributing metabolic and environmental factors, making the uncovering of precise basis of these disorders exceedingly difficult.

Several Mendelian heritable disorders depict phenotypic similarities with acquired forms of metastatic or dystrophic calcification, and many of these conditions serve as genetically controlled models to study various facets of pathological mineralization [5]. Such conditions include pseudoxanthoma elasticum, which demonstrates mineralization in the skin, eyes and the cardiovascular system, as a result of mutations in the *ABCC6* gene [6]. Another very severe ectopic calcification disorder affecting arterial blood vessels and depicting joint and spine ossification is generalized arterial calcification of infancy (GACI), harboring mutations in the *ENPP1* gene [7, 8].

Mouse models corresponding to several heritable ectopic mineralization disorders have been extremely helpful in identifying critical pathways involved. In addition, these model systems have provided evidence of intricate pro-mineralization/anti-mineralization networks in peripheral connective tissues necessary to maintain normal homeostasis preventing ectopic mineralization [5]. While the focus on characterization of these mice has been on mineralization affecting the skin and the cardiovascular system, less attention has been made on ectopic mineralization of cartilage and periarticular tendons and ligaments, major issues encountered in various orthopedic disorders.

One of the recently characterized mice mimicking GACI is the *Enpp1^asj-2J^* mouse (referred to hereon as *asj-2J* mouse). This mouse arose spontaneously in a large-scale production colony of BALB/cJ mice at The Jackson Laboratory [9]. These mutant mice develop abnormal forepaw position and gait due to stiffening of the joints, a phenotype similar to a previously characterized *asj* (“ages with stiffened joints”) mouse harboring p.V246D mutation in the *Enpp1* gene [10]. Therefore, this mutant mouse was named as *asj-2J* being allelic to *asj* mouse. The *asj-2J* mice carry a large, 40,035 bp, deletion from intron 1 to 3′ UTR of the *Enpp1* gene, coupled with a 74 bp insertion [9]. Plasma PPi concentration and PPi/Pi ratio was significantly reduced in homozygous *asj-2J* mice. As a consequence of this spontaneous mutation, extensive mineralization of the arterial vasculature and the dermal sheath of vibrissae was demonstrated by a combination of histopathology with calcium-specific stains, direct chemical assay of calcium, and microcomputed tomography. We have now further characterized the *asj-2J* mouse with particular focus on mineralization of cartilage and collagen-rich ligaments and tendons, with clinical relevance to orthopedic conditions.

## RESULTS

### Experimental design

Previous studies demonstrated that feeding *asj-2J* mice with “acceleration diet”, enriched in phosphate (2x) and reduced in magnesium (20%) content in comparison to control diet, results in acceleration of the ectopic mineralization [9]. We, therefore, placed *Enpp1^+/+^*, *Enpp1^+/asj-2J^ and Enpp1^asj-2J^* mice on either a standard control rodent diet or acceleration diet. Two experimental designs were utilized in different groups of mice (Table 1). In the first set of experiments (Set 1), 12-week-old *asj-2J* mice were examined for ectopic mineralization and subjected to blood analysis, in comparison to wild type and heterozygote littermates of the same age. To determine the onset of mineralization on these two different diets, a second set of homozygous *asj-2J* mice was examined at different earlier time points (Set 2).

**Table 1:** Experimental design for analysis of *asj-2J* mice

Group	Genotype	Age (weeks)	Number of mice examined (female, male)
**Set 1:**			
Normal diet	*Enpp1*^+/+^	12	4, 3
Normal diet	*Enpp1^+/asj-2J^*	12	3, 5
Normal diet	*Enpp1^asj-2J^*	12	2, 3
Acceleration diet	*Enpp1*^+/+^	12	5, 4
Acceleration diet	*Enpp1^+/asj-2J^*	12	3, 3
Acceleration diet	*Enpp1^asj-2J^*	12	4, 2
**Set 2:**			
Normal diet	*Enpp1^asj-2J^*	6	1, 0
Normal diet	*Enpp1^asj-2J^*	7	0, 1
Normal diet	*Enpp1^asj-2J^*	9	1, 0
Acceleration diet	*Enpp1^asj-2J^*	4	1, 1
Acceleration diet	*Enpp1^asj-2J^*	5	2, 1
Acceleration diet	*Enpp1^asj-2J^*	6	1, 0

The mice were maintained on normal or acceleration diet throughout the experiments and sacrificed at different time points as indicated for analysis of ectopic mineralization phenotypes.

### Histopathologic evaluation of mineralization

**Figure 1:**
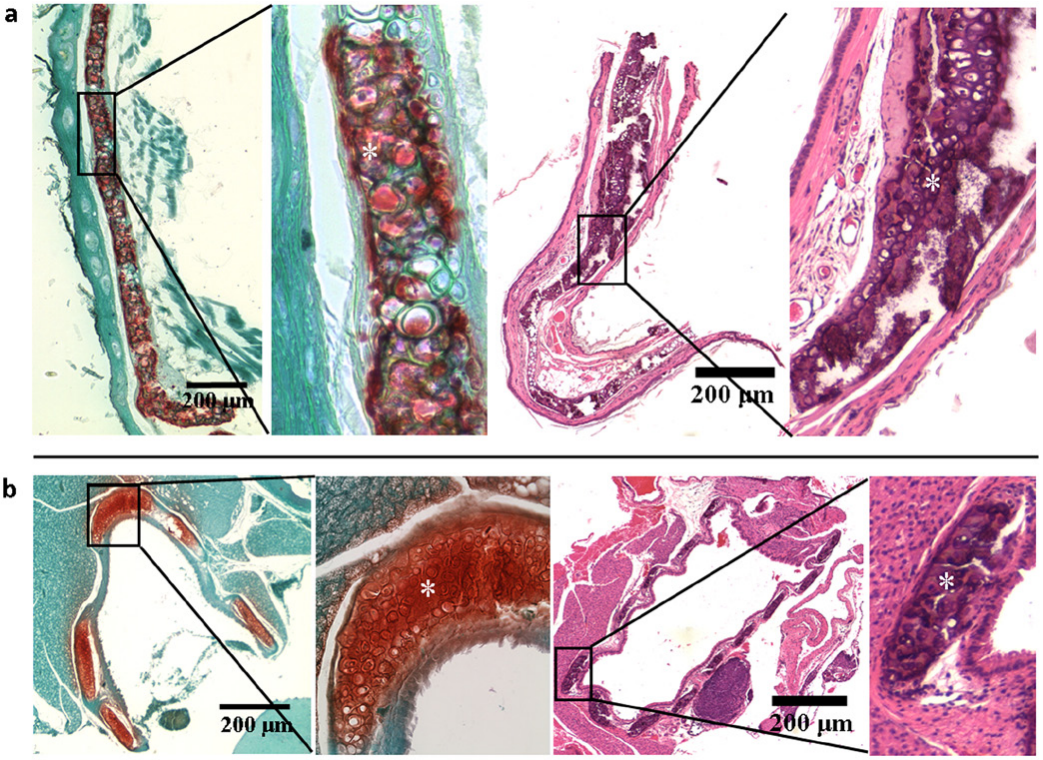
Extensive mineralization of elastic and hyaline cartilages in the *asj-2J* mice at 12 weeks of age (white asterisk) Alizarin red staining (left panel) and H&E staining (right panel) reveal extensive mineralization in the elastic cartilage of outer ear (**a**) and hyaline cartilage of trachea (**b**) of *asj-2J* mice.

Three types of cartilage, hyaline, elastic, and fibrocartilage, with different connective tissue composition and organ distribution were analyzed histologically [11]. Histopathological examination of the outer ear and trachea in formalin fixed paraffin sections with Alizarin red and H&E staining procedures revealed extensive calcification of elastic cartilage and hyaline cartilage, respectively, in *asj-2J* mice at 12 weeks of age when kept on normal or acceleration diet (Figure 1).

**Figure 2:**
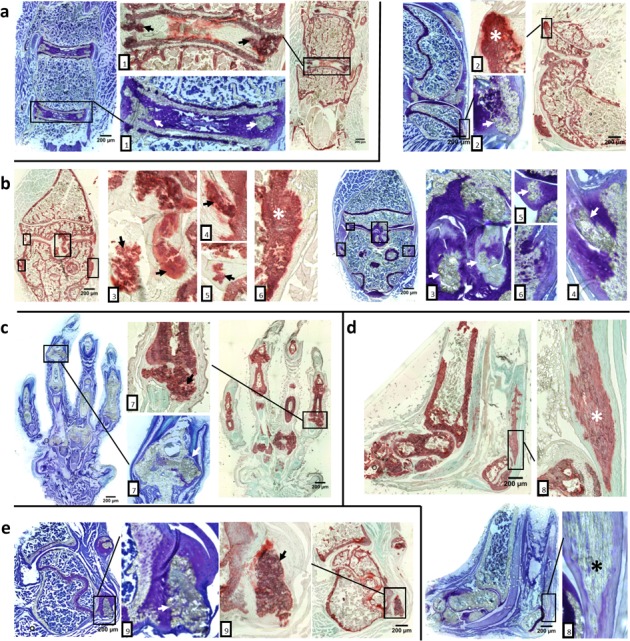
Aberrant cartilage and ligament mineralization in *asj-2J* mice at 12 weeks of age Toluidine Blue O staining (blue & purple) and Alizarin red staining (red & green) reveal extensive cartilage and ligament mineralization in the intervertebral disc, knee, foot, ankle and shoulder of *asj-2J* mice, marked by arrow or asterisk. **a.** Coronal sections of spine show mineralization in intervertebral discs and posterior longitudinal ligament (1). **b.** Coronal and sagittal sections of the knee show mineralized in patellar tendons (2), posterior/anterior cruciate ligaments (3), meniscus (5) and lateral/medial collateral ligaments (4,6). **c.** Coronal sections of the foot show the hypertrophied mineralized joints (7). **d.** Sagittal plane of the ankle shows the Achilles tendon mineralization (8). **e.** Coronal sections of shoulder show supraspinatus tendon mineralization (9).

Assessment of mineralization in articular cartilage and fibrocartilage as well as tendons and ligaments which are attached to long bones was technically difficult using regular fixation and sectioning procedures due to inability to section mineralized tissues. Therefore, we used a cryo-histological method without decalcification of bones [12, 13]. This method is based on the use of cryotape that adheres to the tissue section, which then allows for sectioning of calcified tissues while maintaining tissue morphology. Coronal sections of spine, knee, foot and shoulder were obtained with this methodology. Toluidine Blue O or Alizarin red staining showed aberrant cartilage, tendon and ligament mineralization in intervertebral discs, patellar tendons, meniscii, and supraspinatus tendons of 12-week-old *asj-2J* mice (Figure 2). Toluidine Blue O and Alizarin red staining of sagittal sections of the knee and ankle showed posterior/anterior mineralization of cruciate ligaments, meniscus, lateral/medial collateral ligaments and Achilles tendon in 12-week-old *asj-2J* mice (Figure 2).

Histopathological examination also indicated that the degree of tissue mineralization in *asj-2J* homozygous mice placed on the acceleration diet was higher than in the corresponding mice kept on control diet. Moreover, no mineralization of the cartilage, tendon, and ligament could be noted in wild type and *asj-2J* heterozygous mice at 12 weeks of age (data not shown).

**Figure 3:**
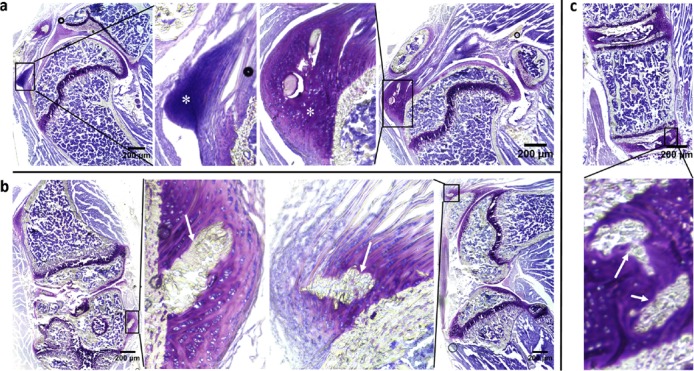
Aberrant fibrocartilage and presence of osteophytes preceding mineralization in *asj-2J* mice at 6 weeks of age (white asterisks and arrows) Toluidine Blue O staining. **a.** Coronal section of the shoulder region shows large amount of fibrocartilage on the surface of supraspinatus tendon. **b.** Coronal/sagittal section of knee shows aberrant osteophytes in joint ligament. **c.** Coronal section of spine shows aberrant osteophytes in intervertebral disc.

While ectopic mineralization was noted in *asj-2J* mice both on control and acceleration diet at 12 weeks of age (Set 1), we next investigated the early changes of cartilage prior to overt ectopic mineralization and the approximate time of onset of mineralization in these mice (Set 2). Mineralization of the cartilage and ligaments was first noted at around 9 weeks of age when *asj-2J* mice were kept on control diet, but changes of cartilage were present as early as at 6 weeks when these mice were kept on the acceleration diet. As shown in Figure 3a, Toluidine Blue O staining revealed large amounts of fibrocartilage on the surface of supraspinatus tendon consisting of unmineralized fibrochondrocytes. These cells mature by becoming hypertrophic towards mineralization. Following this stage of maturation, the mature fibrocartilage is invaded by ectopic immature osteophytes in joint ligaments and intervertebral discs in *asj-2J* mice (Figure 3b, 3c). The immature osteophytes will develop into more mature osteophytes with bone when the mice age.

### Analysis of bone formation and resorption

**Figure 4:**
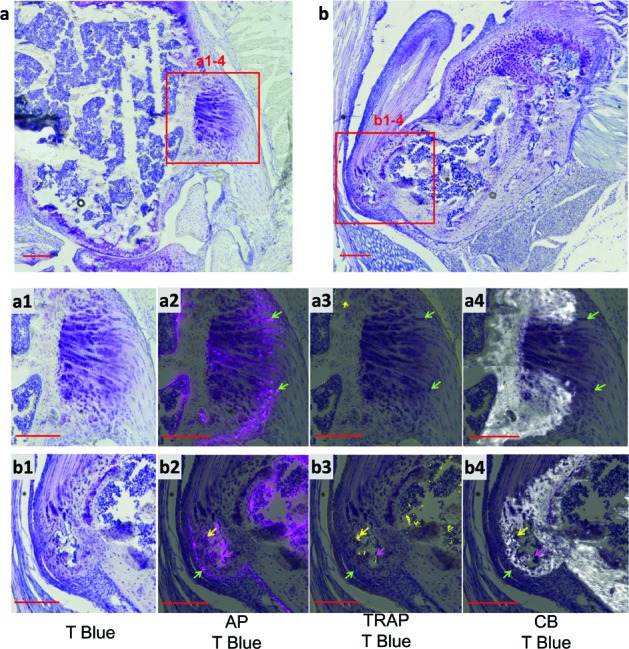
Osteophytes in *asj-2J* mice at 12 weeks of age display AP (pink) and TRAP (yellow) activities in the lateral collateral ligament in the knee (**a)** and the supraspinatus tendon in the shoulder (**b)** AP positive fibrocartilage cells (green arrows) were seen in the unmineralized regions (non-CB) of the shoulder osteophyte and in the less mature knee osteophyte. The shoulder osteophyte was more mature with TRAP positive osteoclasts (yellow arrows) and AP positive osteoblasts (pink arrows) lining the bone surfaces in the interior of the osteophyte. T Blue, Toluidine Blue O stain; AP, alkaline phosphatase; TRAP, tartrate-resistant acid phosphatase; CB, Calcein Blue stain for mineral. Scale bar = 200μm.

Alkaline phosphatase (AP) and tartrate-resistant acid phosphatase (TRAP) activities, reflecting bone formation and bone resorption [14], respectively, were analyzed at the lateral and collateral ligament in the knee and the supraspinatus tendon in the shoulder, sites of ectopic mineralization in the *asj-2J* mice. AP positive osteoblasts and fibrocartilage cells were seen on frozen sections in mineralized osteophytes. Specifically, AP positive osteoblasts line the bone surfaces in the interior of the osteophyte, while AP positive fibrocartilage cells were also seen in the unmineralized regions of osteophyte. TRAP activity, a marker of osteoclasts of bone resorption, could be observed in more mature osteophytes near the supraspinatus tendon in the shoulder, but not in less mature osteophytes in the knee (Figure 4). The positive AP and TRAP stains suggested that bone formation and bone resorption were active processes at the sites of cartilage, tendon and ligament leading to ectopic mineralization.

We further examined AP and TRAP activities in serum of 12-week-old *asj-2J* mice by a colorimetric method. The results indicated that serum TRAP activity was not altered; however, there was a significant increase in AP serum activity in *asj-2J* mice in comparison to the wild type littermates (Table 2), indicating increased bone formation rate in these mice.

**Table 2:** Ca, PPi/Pi ratio, TRAP and AP enzymatic activities, and CRP levels in serum/plasma of mice maintained on a normal rodent diet

Parameter	Concentration/activity (mean ±SEM)		
	*Enpp1*^+/+^ *(n = 8)*	*Enpp1^+/asj-2J^ (n = 11)*	*Enpp1^asj-2J^ (n = 10)*
Ca (mg/dL)	9.38 ± 0.18	9.52 ± 0.15	9.41 ± 0.17
PPi/Pi ratio (x 1,000)	4.22 ± 0.74	2.96 ± 0.38	0.88 ± 0.12*
TRAP activity (U/mL)	0.05 ±0.01	0.05 ±0.007	0.06 ±0.01
AP activity (U/L)	23.63 ± 1.77	23.59 ± 2.02	42.53 ± 1.64**
CRP (ng/μL)	2.70 ± 0.24	-	2.37 ± 0.25

Blood samples were collected by cardiac puncture at 12 weeks of age and Ca, Pi, CRP concentration, and TRAP and AP activities were determined in serum. PPi levels were measured in heparinized plasma. Statistical significance in comparison to *Enpp1*^+/+^ mice:

* *P* < 0.001

** *P* < 0.0001.

### Serum analysis

To examine potential inflammation in *asj-2J* mice, serum C-reactive protein (CRP) levels in 12-week-old *asj-2J* mice and their wild type counterparts were determined by an ELISA. There was no significant difference between these two groups (Table 2).

The *asj-2J* mice were further characterized by analyzing the calcium levels and the PPi/Pi ratio in plasma/serum when kept on normal diet. As shown in Table 2 and reported previously [9], serum calcium levels were not altered, but PPi/Pi ratio was significantly reduced, indicating perturbed homeostasis promoting ectopic mineralization in these mice.

## DISCUSSION

### Connective tissue biochemistry and pathology of cartilage, tendons and ligaments

#### Cartilage

Cartilage, flexible connective tissue providing structural integrity to a number of tissues, is derived from the mesoderm layer. There are three broad categories of cartilage (elastic cartilage, hyaline cartilage and fibrocartilage) with different extracellular matrix composition and distinct tissue distribution. Elastic cartilage is present in the outer ear, Eustachian tube and epiglottis, the principal component being elastin. Fibrocartilage is present in meniscii, intervertebral discs, and tendon/ligament entheses, and it consists of a mixture of type I and II collagens and proteoglycans. Hyaline cartilage is a highly specialized connective tissue in diarthrodial joints with the principal function being to provide a smooth surface for articulation and to facilitate movement with low frictional coefficient. Consequently, injury to articular cartilage can cause significant musculoskeletal morbidity resulting in osteoarthritis [15, 16]. In some cases, osteoarthritis is facilitated by calcification of the cartilage, resulting in rupture and detachment of the articular cartilage as a result of trauma. Thus, cartilage mineralization can have serious consequences with little, if any, regenerative repair capacity, often necessitating joint replacement of knees or hips.

#### Tendons

Of the millions of musculoskeletal injuries reported in the United States per year, roughly 50% of cases involve injuries to the soft tissues, including tendons and ligaments [17]. Tendons consist of fibrous connective tissue composed primarily of densely packed collagen fiber bundles aligned parallel to the longitudinal tendon axis and surrounded by a tendon sheath. Collagen, predominantly type I collagen, constitutes ∼75% of the dry weight of tendon [18]. Lacerations, ruptures or inflammation to the tendon cause marked morbidity and have a major impact on work and recreational activities of the affected individuals. Tendon repair and regenerative processes are slow and often lead to extensive scarring and adhesions.

#### Ligaments

Skeletal ligaments are dense bands of collagenous tissue spanning joints to become anchored to the bones [19]. One of the main functions of ligaments is to mechanically stabilize joints during normal range of motion. The major forms of trauma to the ligaments result in ruptures which depending on the severity and location may or may not heal spontaneously. The healing tissues leads to scar formation and major changes in the overall composition, tissue architecture and function from the original ligament [20]. Mineralization of ligaments can also be noted in a number of clinical situations, often as a sequela to inflammation. Thus, trauma and inflammation can cause lesions to the ligaments and tendons, often resulting in dysfunction and chronic clinical sequela.

### Mineralization of cartilage, tendons and ligaments in *asj-2J* mice

A spontaneous mutant mouse, *asj-2J*, was recently identified to develop extensive soft tissue mineralization, with the primary focus of the previous studies being on the skin and cardiovascular system [9]. Early observations in allelic series of single gene mutations in *Enpp1* in mouse models suggested that there is mineralization also affecting joints and perispinal ligaments [21-24]. In this study, we examined the *asj-2J* mice for cartilage and collagen-rich tendon and ligament mineralization first with standard histopathologic approaches. These studies demonstrated extensive mineralization of cartilages in the outer ear (elastic cartilage) and the trachea (hyaline cartilage). However, examination of fibrocartilage in the joints and mineralization in periarticular tendons and ligaments in close proximity to the bones posed technical difficulties due to our initial inability to section mineralized bone without perturbing the microarchitecture. For this reason, we resorted to the use of a novel, recently developed cryo-sectioning technique, which allows sectioning of mineralized tissues without decalcification [12, 13]. These sections retain their morphology and can be readily examined by staining with Toluidine Blue O and Alizarin red. The results demonstrated extensive mineralization of articular cartilages as well as adjacent ligaments in the knees and Achilles tendon in 12-week-old *asj-2J* mice. The mineralization process was accompanied with positive alkaline phosphatase and tartrate-resistant acid phosphatase activities on the frozen sections at the sites of ectopic mineralization. These mice also demonstrated increased alkaline phosphatase activity in their serum thus explaining the formation of mineral deposits in soft connective tissues.

A number of mouse models with ENPP1 deficiency have been previously reported [21-24], and one of them, *ttw* (tiptoe walking mouse), has been reported to demonstrate mineralization of posterior longitudinal ligament of the spine [22]. This naturally occurring mouse is caused by a nonsense mutation p.Gly568* in the *Enpp1* gene and is, therefore, similar to *asj-2J* mouse. The mineralization process in these mice is apparently elicited by reduced PPi/Pi ratio. Under normal homeostatic conditions, a certain level of PPi is required to prevent precipitation of calcium and phosphate in the form of hydroxyapatite in soft tissues [25]. The *asj-2J* mice are homozygous for a large deletion/insertion mutation in the *Enpp1* gene, and as a result of absent ENPP1 activity, the plasma levels of PPi were reduced in these mice to about 21% of the control mice [9]. Thus, the *asj-2J* mice serve as a genetic model of ectopic soft tissue mineralization. It should be noted that this mineralization process could be accelerated by keeping the mice on a so-called “acceleration diet”, enriched in phosphate and reduced in magnesium content. Thus, the ectopic mineralization in these mice reflects interactions of several factors, including genetic background and diet, at the genome/environment interface.

The *asj-2J* mouse serves as a model for GACI by demonstrating excessive vascular mineralization. Evaluation of the presence of mineralization of cartilage, ligaments and tendons in GACI patients is difficult because these patients frequently die within a few months of life due to vascular complications. However, some of these individuals survive and abnormal calcifications of ear cartilage and cervical fusions have been reported [26]. Finally, it should be noted that *asj-2J* mouse has similarities with *ttw* mouse which serves as a model of a relatively common human disease, ossification of the posterior longitudinal ligament of the spine (OPLL) [27, 28]. Thus, these mouse models serve as a platform to develop novel treatments for these, currently intractable, ectopic mineralization disorders.

## MATERIALS AND METHODS

### Experimental procedures

#### Animals and diet

*Enpp1^asj-2J^* mice (*asj-2J* mice) on a BALB/cJ background were obtained from The Jackson Laboratory (Bar Harbor, ME). *Enpp1^+/+^* as well as heterozygous and homozygous *asj-2J* mutant mice were generated from heterozygous mattings. Mice were genotyped and maintained in a climate-controlled environment either on normal laboratory diet (Laboratory Autoclavable Rodent Diet 5010; PMI Nutritional International, St. Louis, MO) or fed an ‘acceleration diet’ (Rodent diet TD.00442, Harlan Teklad, Madison, WI), which we have previously shown to accelerate the ectopic mineralization in *asj-2J* mice [9]; this diet is enriched in phosphorus (2x) and has reduced magnesium (20%) content. The groups of mice characterized by genotype and dietary treatment are described in Table 1. All animal experiments were approved by Institutional Animal Care and Use Committee of Thomas Jefferson University. Proper handling and care were followed according to the Animal Welfare Policies of the Public Health Service of the USA.

### Histological analysis of soft connective tissues

Histological analysis was performed on the outer ears and trachea of *Enpp1*^+/+^, *Enpp1*^+/^*^asj-2J^*, and *Enpp1^asj-2J^* mice. Tissues were fixed in 10% phosphate-buffered formalin, routinely processed, and embedded in paraffin. Tissues were sectioned (7 μm) and stained with Alizarin red and Hematoxylin & Eosin (H&E) using standard procedures. After staining, the samples were examined under light microscopy for mineralization.

### Cryohistological analysis of bone and cartilage

Forelimbs, hind limbs and lumbar vertebra from euthanized mice were fixed in 10% phosphate-buffered formalin for 2 days at 4°C, transferred to 30% sucrose in PBS overnight at 4°C, and then embedded in O.C.T. Compound (Tissue-Tek, Sakura, Japan). The knee was cut in the sagittal and coronal planes to capture articular cartilage and menisci. The ankle was cut in the sagittal plane, the shoulder and foot were cut in the coronal plane to investigate the articular cartilage. The lumbar vertebrae were cut in sagittal and coronal planes to investigate fibrocartilage in intervertebral discs. All sections were made from undercalcified joints using cryofilm IIC tape (Section Lab Co. Ltd, Hiroshima, Japan), which maintains morphology of mineralized sections. The taped sections were glued to microscope slides, with tissue side up, using UV adhesive glue (Norland Optical Adhesive 63, Norland Products Inc., Cranbury, NJ) and rehydrated prior to staining [12].

### Staining and imaging

Because the cryofilm tape adheres to the tissue and allows for the coverslip to be removed between imaging steps without damaging the section, each section was stained up to 4 times. The order of staining included Calcein Blue, Tartrate-resistant acid phosphatase (TRAP), Alkaline Phosphatase (AP), and Toluidine Blue O staining, on the same section.

#### Calcein Blue staining

Slides were stained with calcein blue to label mineral deposition.

#### TRAP staining

Using 0.92% sodium acetate anhydrous and 1.14% sodium tartrate dibasic dehydrate make fresh TRAP reaction buffer, adjust pH to 4.2. Sections were incubated in TRAP buffer for 10 min and then incubated with Elf97 substrate (Life Tech, Grand Island, NY) in buffer for 5 min under UV light. The Elf97 substrate generates a yellow fluorescent signal when cleaved by TRAP. The TRAP signal was imaged using Zeiss Axio Scan Z1 scanning microscope (Jena, Germany).

#### AP staining

After TRAP staining, the same slides were placed in PBS to remove cover slips. Sections were incubated in AP buffer (100 mM Tris, pH 9.5, 50 mM MgCl_2_, 200 mM NaCl) for 10 min and then incubated with AP substrate, 1x Naphthol AS-MX phosphate, 1x Fast red (Sigma Aldrich, St. Louis, MO) for 5 min at room temperature. The AP signal was imaged using a TRITC filter (Chroma Technology Corp., Bellows Falls, VT).

#### Toluidine Blue O staining

The same slides were stained with 0.025% Toluidine Blue O, and re-imaged.

### Quantification of calcium and phosphate

The calcium content in serum was determined colorimetrically by the σ-cresolphthalein complexone method (Calcium (CPC) Liquicolor; Stanbio Laboratory, Boerne, TX). The phosphate concentration of serum was determined with Malachite Green Phosphate Assay kit (BioAssay Systems, Hayward, CA).

### Inorganic pyrophosphate assay

Whole blood was collected into heparin coated blood collection tubes to separate plasma from cells by centrifugation. The plasma was collected by filtration through a Centrisart I 300-kDa mass cutoff filter (Sartorius, New York, NY), and stored at −80°C until further processing. The concentration of inorganic pyrophosphate (PPi) was determined in plasma with an enzymatic assay using uridine-diphosphoglucose (UDPG) pyrophosphorylase, with modifications, as described previously [10].

### TRAP and AP enzymatic activities and C-reactive protein (CRP) level

Whole blood was collected by cardiac puncture and serum was separated from whole blood by centrifugation, 2,200 x g at 4°C for 15 min. The TRAP enzyme activity in serum was determined colorimetrically by the TRAP Assay Kit (TaKaRa, Kusatsu, Japan). The AP enzyme activity in serum was determined colorimetrically by the Alkaline Phosphatase Assay Kit (Abcam, Cambridge, MA). The serum levels of CRP were tested by Mouse C-Reactive Protein (CRP) ELISA kit (Life Diagnostics, West Chester, PA).

### Statistical analysis

Statistical analyses were performed using two-sided Kruskal-Wallis nonparametric test and *p* < 0.01 was considered to indicate a statistically significant difference. All statistical computations were completed using SPSS (version 15.0, Chicago, IL) and GraphPad PRISM (version 5.01, San Diego, CA).

## Abbreviations

GACI: generalized arterial calcification of infancy
asj: ages with stiffened joints
AP: alkaline phosphatase
TRAP: tartrate-resistant acid phosphatase
PPi: pyrophosphate
Pi: phosphate
